# Four new species of the primitively segmented spider genus *Qiongthela* from Hainan island, China (Mesothelae, Liphistiidae)

**DOI:** 10.3897/zookeys.714.19858

**Published:** 2017-11-06

**Authors:** Xin Xu, Fengxiang Liu, Matjaž Kuntner, Daiqin Li

**Affiliations:** 1 College of Life Sciences, Hunan Normal University, Changsha, Hunan, China; 2 Centre for Behavioural Ecology and Evolution (CBEE), College of Life Sciences, Hubei University, Wuhan, Hubei, China; 3 Institute of Biology, Research Centre of the Slovenian Academy of Sciences and Arts, Ljubljana, Slovenia; 4 Department of Entomology, National Museum of Natural History, Smithsonian Institution, Washington, D.C, USA; 5 Department of Biological Sciences, National University of Singapore, 14 Science Drive 4, Singapore 117543

**Keywords:** China, primitively segmented spiders, *Qiongthela*, taxonomy, trapdoor spiders

## Abstract

Four species of the primitively segmented spider genus *Qiongthela* Xu & Kuntner, 2015 collected from Hainan Island, China are diagnosed and described as new to science based on their genital morphology: *Q. bawang* **sp. n.** (♀), *Q. jianfeng* **sp. n.** (♂♀), *Q. yini* **sp. n.** (♀), *Q. wuzhi* **sp. n.** (♂♀). Together with the type species of *Qiongthela*, *Q. baishensis* Xu, 2015, five species are found and described from Hainan, China, and seven species are now known in the genus.

## Introduction

The primitively segmented spider family Liphistiidae (Araneae, Mesothelae), an ancient lineage that retains plesiomorphic arachnid traits such as the abdominal tergites and spinnerets located in the middle of abdominal venter, currently contains 101 species in eight genera (World Spider Catalog 2017; Xu et al. 2015a, b). These extant species are restricted to Southeast and East Asia and display an extremely high level of endemism and disjunct distribution (Xu et al. 2015a, b, 2016). They are divided into two subfamilies, Heptathelinae and Liphistiinae (Xu et al. 2015a, b). While the subfamily Liphistiinae contains a single genus (*Liphistius* Schiödte, 1849) with species in Southeast Asia (Sumatra, Laos, Malaysia, Myanmar, Thailand), Heptathelinae has seven genera. Two of them (*Heptathela* Kishida, 1923 and *Ryuthela* Haupt, 1983) are confined to Japan (Ryukus islands and Kyushu), and the other five are distributed between mainland China and Vietnam: *Ganthela* Xu & Kuntner, 2015, *Qiongthela* Xu & Kuntner, 2015, *Sinothela* Haupt, 2003, *Songthela* Ono, 2000 and *Vinathela* Ono, 2000) (World Spider Catalog 2017; Xu et al. 2015a, b). Since the genus level revision of the family (Xu et al. 2015a, b), species-level revisions have been proposed for two genera, *Ganthela* (Xu et al. 2015c) and *Ryuthela* (Xu et al. 2017).

In this study, we focus on the genus *Qiongthela* from Hainan Island, China, located in the transitional zone between tropical and temperate zones in the South China sea. *Qiongthela* was diagnosed in 2015 (Xu et al. 2015a, b), but so far, only three *Qiongthela* species have been named: *Q. baishensis* Xu, 2015, the type species from Hainan, and *Q. australis* (Ono, 2002) and *Q. nui* (Schwendinger & Ono, 2011) from southern Vietnam (Xu et al. 2015b). Even though no molecular data were available for the two Vietnamese species of *Qiongthela* in our previous studies (Xu et al. 2015a), the genital morphology confirms their inclusion in *Qiongthela* (Ono 2002; Schwendinger and Ono 2011; Xu et al. 2015b). Here, four new species of *Qiongthela* collected from Hainan are diagnosed and described. Their taxonomy is based on male and female genital morphology combined with the results from prior phylogenetic analyses, which support the four new species (fig. 2 in Xu et al. 2015a).

## Materials and methods

**Figure 1.**
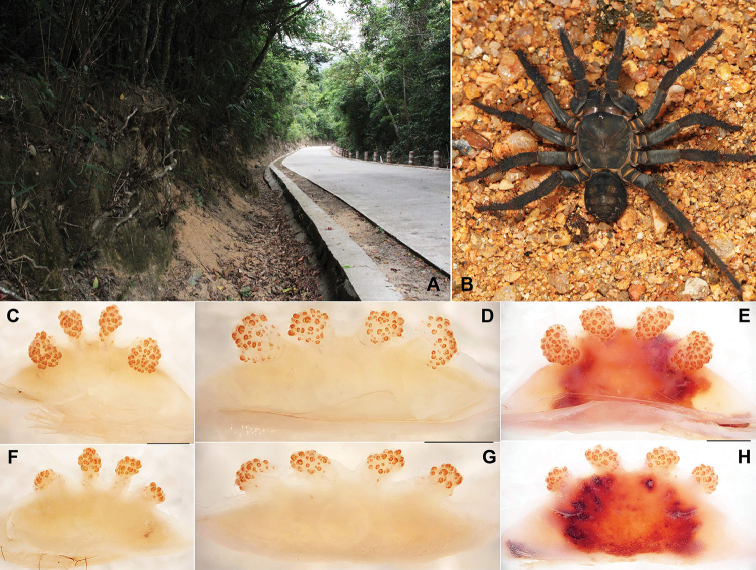
Macrohabitat, general somatic morphology and genital anatomy of *Qiongthela bawang* sp. n. **A** Macrohabitat of *Qiongthela bawang* sp. n. at the type locality **B** Female (XUX-2012-097) **C–E** vulva dorsal view **F–H** vulva ventral view **C, F** (XUX-2014-012) **D, G** (XUX-2012-097) **E, H** (XUX-2011-001). Scale bars: 0.5 mm.

**Figure 2.**
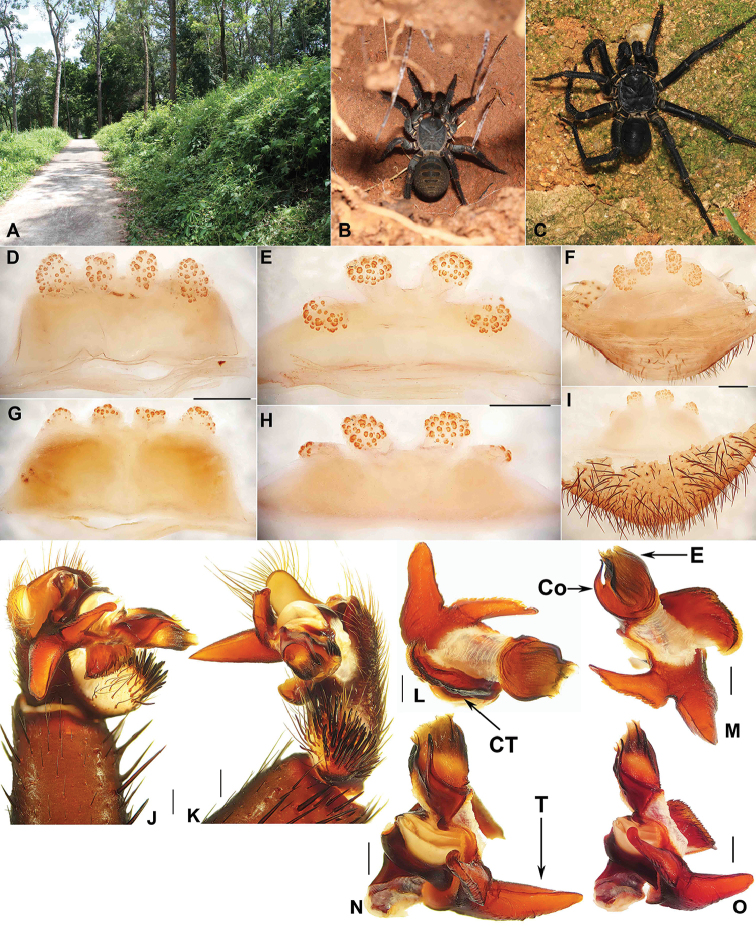
Macrohabitat, general somatic morphology and genital anatomy of *Qiongthela jianfeng* sp. n. **A** Macrohabitat of *Qiongthela jianfeng* sp. n. at the Forest Research Station **B** Female (XUX-2012-098) **C** male (XUX-2012-107) **D–F** vulva dorsal view **G–I** vulva ventral view **J** palp ventral view **K** palp retrolateral view **L–O** palp distal view **D, G** (XUX-2014-009) **E, H** (XUX-2014-002) **F, I** (XUX-2012-098) **J–K** (XUX-2014-004) **L–O** (XUX-2014-005). Scale bars: 0.5 mm.

All *Qiongthela* specimens in this study were collected at the roadside of forest (Figs 1A, 2A, 4A). They were collected alive and fixed in absolute alcohol if they were adults. The subadults were brought back to the laboratory and reared until they reached maturation. All specimens were then preserved in 80% ethanol after the right four legs were removed for molecular work.

Specimens were studied using an Olympus SZX16 stereomicroscope. Anatomical details were examined and photographed with on Olympus BX53 compound microscope and a Canon 7D camera. Genitalia were cleared in boiling KOH for a few minutes to dissolve soft tissues. All the specimens were deposited at the Centre for Behavioural Ecology and Evolution (CBEE), College of Life Sciences, Hubei University, Wuhan, China. All lengths are given in millimetres. Leg and palp measurements are given in the following order: total length (femur + patella + tibia + metatarsus + tarsus).

Abbreviations used:

**ALE** anterior lateral eyes,

**AME** anterior median eyes,

**BL** body length,

**CL** carapace length,

**Co** conductor,

**CT** contrategulum,

**CW** carapace width,

**E** embolus,

**OL** opisthosoma length,

**OW** opisthosoma width,

**PLE** posterior lateral eyes,

**PME** posterior median eyes,

**RC** receptacular cluster,

**T** tegulum.

## Taxonomy

### Genus Qiongthela

Taxon classificationAnimaliaAraneaeLiphistiidae

Xu & Kuntner, 2015

#### Type species.

*Qiongthela baishensis* Xu, 2015

#### Diagnosis.

*Qiongthela* males can be distinguished from all other Heptathelinae genera by the blade-like conductor narrowing to a slightly hooked apex (Fig. 2M–O; Fig. 4F–J), and by the tegulum with two distinct apophyses (Fig. 2L–O; Fig. 4F–J). The females differ from all other Heptathelinae genera by two paired receptacular clusters, all with numerous granula (e.g. Fig. 1C–H) (Xu et al. 2015b).

#### Species composition.

*Q. australis* (Ono, 2002), *Q. nui* (Schwendinger & Ono, 2011), *Q. baishensis* Xu, 2015, *Q. bawang* sp. n., *Q. jianfeng* sp. n., *Q. yini* sp. n., *Q. wuzhi* sp. n.

#### Distribution.

China (Hainan), Vietnam.

### Qiongthela bawang sp. n.

Taxon classificationAnimaliaAraneaeLiphistiidae

http://zoobank.org/154A4D7B-7EF4-411C-8AAD-EE1952264EB5

Fig. 1

#### Holotype.

Female (XUX-2011-001), Bawangling, Changjiang County, Hainan Province, China, 19.04°N, 109.09°E, 657 m, collected 19 June 2011 by D. Li, F. Liu, M. Kuntner and X. Xu, deposited at CBEE, College of Life Sciences, Hubei University, Wuhan, China.

#### Paratypes.

2 females and 2 juveniles (XUX-2012-(094-097)), Bawangling Nature Reserve, Bawangling, Changjiang County, Hainan Province, China, 19.24°N, 109.38°E, 462 m, collected 19–20 July 2012 by D. Li, F. Liu and X. Xu; 1 female (XUX-2014-012) collected at the same locality, 19.03°N, 109.10°E, 711 m, collected 23 March 2014 by F. Liu and C. Xu.

#### Etymology.

The species epithet, a noun in apposition, refers to the type locality.

#### Diagnosis.

Females of the new species differ from *Q. jianfeng* sp. n. by the two pairs of receptacular clusters along the anterior margin of bursa copulatrix, which are visible in both dorsal and ventral views (Fig. 1C-H). It can be distinguished from *Q. baishensis* and *Q. nui* by its receptacular clusters with similar size or the middle pair being slightly smaller than the lateral pair. It also differs from all other *Qiongthela* species in Hainan by its receptacular clusters, all of which have short genital stalks (Fig. 1C-H). The male is unknown. To facilitate future identification of the species, we provided the DNA barcode for the holotype (XUX-2011-001), which is available on GenBank (Genbank accession code KP229897).

#### Description.

Female (Fig. 1B). Carapace dark brown; opisthosoma darker than carapace; sternum narrow; a few long pointed hairs running over ocular mound in a longitudinal row; chelicerae robust with promargin of cheliceral groove containing 9-10 denticles of variable size; legs with strong hairs and spines; opisthosoma with 12 tergites, close to each other, the fifth largest; seven spinnerets. Measurements: BL 15.75-30.35, CL 8.37-15.95, CW 7.88-12.50, OL 7.42-16.50, OW 5.75-15.50; ALE > PLE > PME > AME; palp 24.10 (8.25 + 4.35 + 5.15 + 6.35), leg I 25.80 (8.45 + 4.75 + 5.05 + 5.00 + 2.55), leg II 24.70 (7.65 + 4.50 + 4.85 + 5.00 + 2.70), leg III 26.80 (7.85 + 4.80 + 4.70 + 6.10 + 3.35), leg IV 37.80 (11.00 + 5.85 + 6.95 + 9.45 + 4.55).

Female genitalia. Two pairs of receptacular clusters along the anterior margin of bursa copulatrix, similar size or the middle pair slightly smaller, with short genital stalks (Fig. 1C–D)

#### Distribution.

Hainan (Bawangling), China

### Qiongthela jianfeng sp. n.

Taxon classificationAnimaliaAraneaeLiphistiidae

http://zoobank.org/91D190C7-2C68-4C7A-B957-D6E89E68AF4E

Fig. 2

#### Holotype.

Male (XUX-2014-005), Jianfeng National Forest Park, Jianfeng Town, Ledong County, Hainan Province, China; 18.70°N, 108.84°E, 508 m, collected 20 March 2014 by F. Liu and C. Xu, deposited at CBEE, College of Life Sciences, Hubei University, Wuhan, China.

#### Paratypes.

One female (XUX-2014-002) and one male (XUX-2014-004, matured 2 August 2014 at CBEE, College of Life Sciences, Hubei University) collected at the same locality, 20 March 2014, by F. Liu and C. Xu; 1 male (XUX-2012-107) collected at the same locality, 18.70°N, 108.84°E, 500 m, collected 22 July 2012 by D. Li, F. Liu and X. Xu; 1 female (XUX-2012-098) and 1 male (XUX-2012-100, matured 10 October 2012 at CBEE, College of Life Sciences, Hubei University) collected at the Forest Research Station, Jianfeng Town, Ledong County, Hainan Province, China, 18.70°N, 108.78°E, 145 m, collected 21 July 2012 by D. Li, F. Liu and X. Xu; 1 female (XUX-2014-008) collected at the same locality, collected 21 March 2014 by F. Liu and C. Xu.

#### Etymology.

The species epithet, a noun in apposition, refers to the type locality.

#### Diagnosis.

Males of the new species differ from all other *Qiongthela* species by the semioval apophysis at the basal conductor (Fig. 2N, O), from *Q. wuzhi* sp. n. by a similar rectangular, rather than the basal angle between the two apophyses of tegulum more than 90° (Fig. 2L), by the distal part of contrategulum with two edges, the inner one being dentate and the outer one being sharp (Fig. 2L, M), and by the distal edge of embolus slightly curved (Fig. 2M). Females of the new species differ from all other *Qiongthela* species by the receptaculuar clusters located slightly on the dorsal wall of the bursa copulatrix, especially the lateral pair being indistinct from the dorsal view (Fig. 2D-I). The DNA barcode of the paratype (XUX-2012-107) is available on GenBank (Genbank accession code KP229838) for future identification. The DNA barcodes of the holotype (XUX-2014-005) and paratype (XUX-2012-107) are identical (the K2P distance between the two sequences is zero).

#### Description.

Male (Fig. 2C). Carapace dark; opisthosoma darker than carapace; sternum narrow; a few long pointed hairs running over ocular mound in a longitudinal row; chelicerae robust with promargin of cheliceral groove containing nine denticles of variable size; legs with strong hairs and spines; opisthosoma with 12 tergites, close to each other, the fifth largest; seven spinnerets. Measurements: BL 17.91-22.00, CL 7.35-12.2,0 CW 7.18-11.00, OL 10.35-10.00, OW 9.07-7.70; ALE > PLE > PME > AME; leg I 37.00 (10.40 + 4.90 + 8.10 + 9.60 + 4.00), leg II 36.95 (10.20 + 4.75 + 7.50 + 10.30 + 4.20), leg III 36.90 (9.70 + 4.80 + 6.80 + 11.00 + 4.60), leg IV 46.80 (12.20 + 5.30 + 9.40 + 14.20 + 5.70).

Palp. The bulb of four specimens of the new species all relatively distorted. Prolateral side of paracymbium unpigmented and unsclerotised, many setae situated at the tip of paracymbium (Fig. 2J, K). Contrategulum with a proximally irregular dentate edge and two distal edges, the inner one dentate (Fig. 2L, M), the outer one sharp (Fig. 2L, M). Tegulum with a long, pointed, distally directed marginal apophysis with a smooth edge, a proximally directed terminal apophysis narrowing to a slightly bent apex, and the dorsal side of terimal apophysis with dentate edge (Fig. 2L, M). Conductor situated ventro-proximal part of embolus, fused with embolus at the basal portion, distal free, narrowing to a slightly hooked apex (Fig. 2M–O). Embolus largely sclerotised, with a wide, flat opening, curved distal edge (Fig. 2M).

Female (Fig. 2B). Colouration of carapace and opisthosoma similar to or lighter than male according to the age of specimens; chelicerae robust with promargin of cheliceral groove with 9-10 strong denticles of variable size; legs furnished with strong hairs and spines; opisthosoma with 12 tergites, similar to male; seven spinnerets. Measurements: BL 14.75-20.00, CL 7.25-11.56, CW 6.20-10.04, OL 7.30-12.00, OW 5.60-9.50; ALE > PLE > PME > AME; palp 12.96 (4.55 + 2.25 + 2.80 + 3.36), leg I 15.47 (4.96 + 2.65 + 2.91 + 3.25 + 1.70), leg II 14.40 (4.55 + 2.30 + 2.65 + 3.25 + 1.65), leg III 15.50 (4.35 + 2.75 + 2.60 + 3.75 + 2.05), leg IV 22.51 (6.36 + 3.20 + 3.95 + 6.15 + 2.85).

Female genitalia. Two pairs of receptacular clusters located slightly on the dorsal wall of the bursa copulatrix, especial the lateral pair indistinct in dorsal view, each receptacular cluster similar size, with or without a genital stalk (Fig. 2D-I).

#### Distribution.

Hainan (Jianfeng), China

### Qiongthela yini sp. n.

Taxon classificationAnimaliaAraneaeLiphistiidae

http://zoobank.org/DD0398BF-75CE-4ED2-9B8A-38615935DD78

Fig. 3

#### Holotype.

Female (XUX-2012-106, matured 18 July 2013 at CBEE, College of Life Sciences, Hubei University), Jianfengling National Forest Park, Jianfeng Town, Ledong County, Hainan Province, China, 18.70°N, 108.86°E, 764 m, collected 22 July 2012 by D. Li, F. Liu and X. Xu, deposited at CBEE, College of Life Sciences, Hubei University, Wuhan, China.

#### Etymology.

The specific epithet honors the arachnologist Changmin Yin, a pioneering liphistiid specialist in China.

#### Diagnosis.

**Figure 3.**
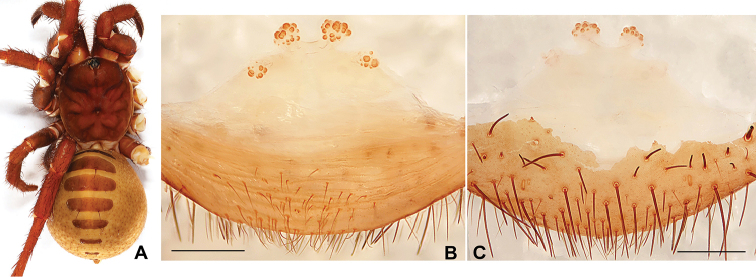
General somatic morphology and genital anatomy of *Qiongthela yini* sp. n. **B** Female (XUX-2012-106) **B** vulva dorsal view **C** vulva ventral view. Scale bars: 0.5 mm.

The female of the new species differs from the other *Qiongthela* species by the receptacular clusters with less granula, the middle pair larger than the lateral pair, the middle pair along the anterior margin of bursa copulatrix, and the lateral pair located on the dorsal wall of the bursa copulatrix (Fig. 3B-C). The male is unknown. The DNA barcode of the holotype (XUX-2012-106) is available on GenBank (Genbank accession code KP229895) for facilitating future identification of the species.

#### Description.

Female (Fig. 3A). Carapace reddish brown in alcohol; opisthosoma light brown; sternum narrow, much longer than wide; a few long pointed hairs running over ocular mound in a longitudinal row; chelicerae robust with promargin of cheliceral groove with ten strong denticles of variable size; legs furnished with strong hairs and spines; opisthosoma with 12 tergites, separated from each other, the first 2-7 larger than others and the fifth largest; seven spinnerets. Measurements: BL 20.50, CL 8.00, CW 7.21, OL 12.40, OW 10.00; ALE > PLE > PME > AME; palp 13.54 (4.70 + 2.43 + 2.80 + 3.61), leg I 15.90 (5.10 + 2.80 + 3.11 + 3.20 + 1.69), leg II 15.35 (4.70 + 2.80 + 2.79 + 3.41 + 1.65), leg III 13.85 (4.35 + 2.10 + 2.60 + 3.00 + 1.80), leg IV 23.71 (7.00 + 3.35 + 4.18 + 6.30 + 2.88).

Female genitalia. The middle receptacular clusters along the anterior margin of bursa copulatrix, the lateral pair located on the dorsal wall of the bursa copulatrix, the middle pair larger than the lateral pair, and the middle pair with short genital stalks (Fig. 3B).

#### Distribution.

Hainan (Jianfeng), China.

#### Remarks.

This new species was found at the Jianfengling National Forest Park as some specimens of *Q. jianfeng* sp. n., but at a higher altitude compared with the latter.

### Qiongthela wuzhi sp. n.

Taxon classificationAnimaliaAraneaeLiphistiidae

http://zoobank.org/19D93B63-0BD5-446F-8E4C-E2058521B02A

Fig. 4

#### Holotype.

Male (XUX-2012-109, matured 4 October 2012 at CBEE, College of Life Sciences, Hubei University), Yongxun Village, Shuiman Town, Wuzhishan City, Hainan Province, China, 18.90°N, 109.63°E, 551 m, collected 25 July 2012 by D. Li, F. Liu and X. Xu, deposited at CBEE, College of Life Sciences, Hubei University, Wuhan, China.

#### Paratypes.

One female (XUX-2012-108) collected at the same locality, collected 25 July 2012 by D. Li, F. Liu and X. Xu.

#### Etymology.

The species epithet, a noun in apposition, refers to the type locality.

#### Diagnosis.

**Figure 4.**
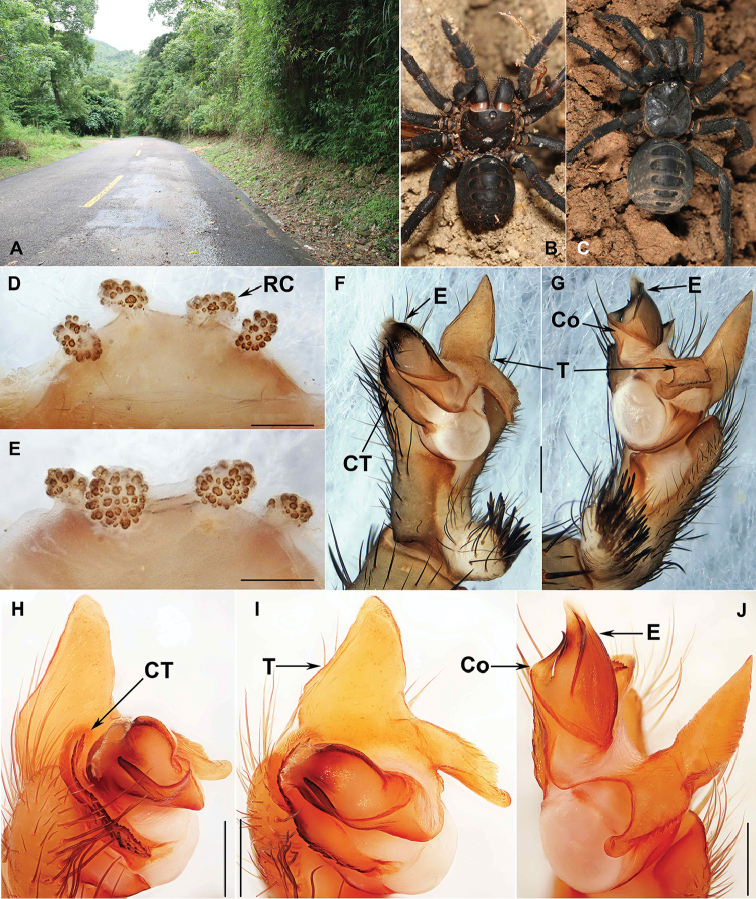
Macrohabitat, general somatic morphology and genital anatomy of *Qiongthela wuzhi* sp. n. **A** Macrohabitat of *Qiongthela wuzhi* sp. n. at the type locality **B** female (XUX-2012-108) **C** male (XUX-2012-109) **D** vulva dorsal view **E** vulva ventral view **F** palp prolateral view **G** palp retrolateral view **H–J** palp distal view. Scale bars: 0.5 mm.

Male of this new species differs from *Q. jianfeng* sp. n. by the contrategulum with three distal edges (Fig. 4H, J), the basal angle between the two apophyses of tegulum more than 90° (Fig. 4I), and the smooth distal margin of embolus (Fig. 4I). It differs from *Q. baishensis*, *Q. australi,s* and *Q. nui* by three distal margins of contrategulum (Fig. 4H, J). Females of this new species can be distinguished from the other species of *Qiongthela* by the receptacular clusters with very short genital stalks, and from *Q. bawang* sp. n. by the irregular shapes of receptacular clusters (Fig. 4D, E). The DNA barcode of the paratype (XUX-2012-108) is available on GenBank (Genbank accession code KP229812) for future identification. The DNA barcodes of the holotype (XUX-2012-109) and paratype (XUX-2012-108) are identical (the K2P distance between the two sequences is zero).

#### Description.

Male (holotype) (Fig. 4C). Carapace black; opisthosoma light brown; sternum narrow, nearly twice as long as wide; a few long pointed hairs running over ocular mound in a longitudinal row; chelicerae robust with promargin of cheliceral groove with eight denticles of variable size; legs furnished with strong hairs and spines; opisthosoma with 12 tergites, the first 2-7 close to each other and larger than others; seven spinnerets. Measurements: BL 14.75, CL 6.20, CW 6.45, OL 9.65, OW 7.45; ALE > PLE > PME > AME; leg I 17.71 (5.03 + 2.52 + 3.86 + 4.50 + 1.80), leg II 17.88 (5.00 + 2.35 + 3.67 + 4.48 + 2.38), leg III 18.87 (4.65 + 2.55 + 3.52 + 5.45 + 2.70), leg IV miss.

Palp. Prolateral side of paracymbium unpigmented and unsclerotised, many setae situated at the tip of paracymbium (Fig. 4F. G). Contrategulum with a proximally irregular dentate edge and three distal edges, the inner one dentate, very short, the middle one dentate, running down to the proximally irregular dentate edge of contrategulum, the outer one sharp, fused with the inner one at the middle portion of the middle edge (Fig. 4H–J). Tegulum with a wide base, pointed, distally directed marginal apophysis with a sharp edge, with a proximally directed terminal apophysis narrowing to a slightly bent apex, and the dorsal side of terminal apophysis with dentate edge (Fig. 4F, G, I, J). Embolus largely sclerotised, with a wide, flat opening, and a sharp distal edge (Fig. 4F–J).

Female (Fig. 4B). Colouration of carapace and opisthosoma reddish dark; female similar to male except larger than male in size; chelicerae robust with promargin of cheliceral groove with 10 strong denticles of variable size; legs furnished with strong hairs and spines; opisthosoma with 12 tergites, tergites larger than that of male; 7 spinnerets. Measurements: BL 20.35, CL 10.35, CW 8.55, OL 11.25, OW 8.65; ALE > PLE > PME > AME; palp 18.28 (6.35 + 3.18 + 4.00 + 4.75), leg I 21.27 (7.05 + 3.75 + 4.12 + 4.48 + 1.87), leg II 20.06 (6.67 + 3.27 + 3.62 + 4.45 + 2.05), leg III 20.82 (6.18 + 3.75 + 3.35 + 5.07 + 2.47), leg IV 30.54 (8.90 + 4.50 + 5.51 + 8.13 + 3.50).

Female genitalia. The two pairs of receptacular clusters along the anterior margin of bursa copulatrix, irregular receptacular clusters with very short genital stalks (Fig. 4D, E).

#### Distribution.

Hainan (Wuzhishan), China.

## Supplementary Material

XML Treatment for Qiongthela

XML Treatment for Qiongthela bawang

XML Treatment for Qiongthela jianfeng

XML Treatment for Qiongthela yini

XML Treatment for Qiongthela wuzhi
